# The Effects of Butyrate on Induced Metabolic-Associated Fatty Liver Disease in Precision-Cut Liver Slices

**DOI:** 10.3390/nu13124203

**Published:** 2021-11-24

**Authors:** Grietje H. Prins, Melany Rios-Morales, Albert Gerding, Dirk-Jan Reijngoud, Peter Olinga, Barbara M. Bakker

**Affiliations:** 1 Department of Pharmaceutical Technology and Biopharmacy, University of Groningen, Groningen Research Institute of Pharmacy, A. Deusinglaan 1, 9713 AV Groningen, The Netherlands; g.g.h.prins@rug.nl; 2 Laboratory of Pediatrics, Center for Liver, Digestive and Metabolic Diseases, University of Groningen, University Medical Center Groningen, A. Deusinglaan 1, 9713 AV Groningen, The Netherlands; m.y.rios.morales@umcg.nl (M.R.-M.); a.gerding@umcg.nl (A.G.); d.j.reijngoud01@umcg.nl (D.-J.R.); 3 Department of Laboratory Medicine, University of Groningen, University Medical Center Groningen, A. Deusinglaan 1, 9713 AV Groningen, The Netherlands

**Keywords:** MAFLD, NAFLD, butyrate, SCFA, steatosis, liver, fatty acid oxidation, fibrosis

## Abstract

Metabolic-associated fatty liver disease (MAFLD) starts with hepatic triglyceride accumulation (steatosis) and can progress to more severe stages such as non-alcoholic steatohepatitis (NASH) and even cirrhosis. Butyrate, and butyrate-producing bacteria, have been suggested to reduce liver steatosis directly and systemically by increasing liver β-oxidation. This study aimed to examine the influence of butyrate directly on the liver in an ex vivo induced MAFLD model. To maintain essential intercellular interactions, precision-cut liver slices (PCLSs) were used. These PCLSs were prepared from male C57BL/6J mice and cultured in varying concentrations of fructose, insulin, palmitic acid and oleic acid, to mimic metabolic syndrome. Dose-dependent triglyceride accumulation was measured after 24 and 48 h of incubation with the different medium compositions. PCLSs viability, as indicated by ATP content, was not affected by medium composition or the butyrate concentration used. Under induced steatotic conditions, butyrate did not prevent triglyceride accumulation. Moreover, it lowered the expression of genes encoding for fatty acid oxidation and only increased C4 related carnitines, which indicate butyrate oxidation. Nevertheless, butyrate lowered the fibrotic response of PCLSs, as shown by reduced gene expression of fibronectin, alpha-smooth muscle actin and osteopontin, and protein levels of type I collagen. These results suggest that in the liver, butyrate alone does not increase lipid β-oxidation directly but might aid in the prevention of MAFLD progression to NASH and cirrhosis.

## 1. Introduction

The first stage of metabolic-associated fatty liver disease (MAFLD, previously referred to as non-alcoholic fatty liver disease) is characterized by hepatic triglyceride accumulation, also known as steatosis [1]. While the pathology has surpassed a global prevalence of 25%, this stage often goes unnoticed in patients [1,2]. It is the progression of the disease to an inflammatory state called non-alcoholic steatohepatitis and beyond, that places the more obvious burden on patients, healthcare and the economy [3,4]. As the hepatic manifestation of obesity, MAFLD is closely related to metabolic syndrome. Despite prevalence of and knowledge about the disease, there is still no appropriate treatment [2,5]. Therefore, potential strategies to ameliorate MAFLD should be investigated.

Consumption of a western diet, which is high in carbohydrates and fatty acids, is often considered to be the cause of metabolic syndrome [6]. This condition is characterized by reduced glucose tolerance, dyslipidaemia and insulin resistance [7]. This leads to an unregulated uptake of saccharides and fatty acids by the liver, by which hepatic lipogenesis is increased and fatty acid oxidation is reduced. This imbalance in lipid metabolism leads to hepatic steatosis [8].

Different from the dietary fatty acids, short-chain fatty acids (SCFA) are the product of fibre fermentation by the gut microbiota. These products, mainly acetate, propionate and butyrate, can be locally metabolised, or absorbed and transported to the liver for further metabolism [9]. High fibre intake has been linked to improvement of metabolic syndrome by reducing body weight, improving insulin sensitivity and increasing energy expenditure [6,10,11].

Accumulating literature describes butyrate as a link between diet and metabolic health of the host. In patients, reduced levels of butyrate are associated with more advanced liver disease [12]. In rodents, a high-fat diet reduced butyrate production and increased levels of inflammation, hepatic steatosis and cholesterol [13]. Also in rodents, oral administration of butyrate or butyrate-producing bacteria decreased hepatic lipogenesis and increased β-oxidation [11] and prevented the onset of MAFLD and progression to NASH [11,14,15,16].

The beneficial effects on the liver of oral butyrate supplementation can be indirect due to inter-organ crosstalk, e.g., by butyrate stimulating GLP-1 secretion from the gut [17], improving the intestinal barrier [16,18], modulating the enterohepatic immune response [19], or by regulating adipose tissue metabolism [10,20]. Another option is that butyrate influences the liver directly, which has been less thoroughly studied. Butyrate can exert its effects in different ways. First, it can increase PPAR dependent signalling which leads to AMP-activated protein kinase (AMPK) phosphorylation, by which metabolism and inflammation are regulated [13,19]. This was shown to increase β-oxidation in HepG2 cells and in ex vivo mouse liver tissue [11]. Secondly, the SCFA can regulate gene transcription via inhibition of histone deacetylase proteins (HDACs) [21,22]. In this way, butyrate was able to enhance GLP-1R protein expression [23] and downregulate NF-κB transcription, which attenuated inflammation and fibrosis [24,25]. Lastly, butyrate can also be used as an energy substrate by the host. Butyrate can enter the mitochondria where it is oxidized to acetyl-CoA. The resulting molecule can be used for ATP production and also as a precursor for lipid synthesis [26,27]. The differential effects butyrate has on the coordination of lipid metabolism towards oxidation or synthesis, and the direct consequences this has on liver inflammation, need to be further addressed.

To investigate the direct effects of butyrate on the liver, studies often use simple cell cultures such as HepG2 cells [28]. In MAFLD development and progression, communication between different cell types is essential [4,29]. Many in vitro models lack the cellular interplay that is required to accurately mimic disease [30]. Precision-cut liver slices (PCLSs) retain the multicellular environment of the liver and have been used to study various diseases [31]. PCLSs have been thoroughly characterized as a fibrosis model [32,33]. A more recent study shows that it is possible to accumulate triglycerides in rat PCLSs, through reduced β-oxidation and increased *de novo* lipogenesis, in a pathophysiological manner [34]. Therefore, the aim of this study was to examine the liver-specific effects of butyrate on lipid metabolism in an ex vivo induced MAFLD-PCLSs model.

## 2. Materials and Methods

### 2.1. Animals

Male C57/BL6 mice (Centrale Dienst Proefdieren, UMCG, Groningen, The Netherlands), aged 9 to 12 weeks, were housed under standard conditions with water and chow ad libitum. Their body weight ranged from 22 to 32 g. Livers from 16 mice were harvested after exsanguination under isoflurane/O_2_ anaesthesia and stored in ice-cold organ preservation solution (University of Wisconsin). Cold ischaemia time was less than one hour. Experiments were approved by the Animal Ethical Committee of the University of Groningen.

### 2.2. Precision-Cut Liver Slices (PCLSs)

PCLSs, with an estimated thickness of ~250 µm, were prepared using a Krumdieck Tissue Slicer (Alabama Research and Development, Munford, AL, USA). PCLSs were cultured in different media at 37 °C under continuous supply of 80% O_2_ and 5% CO_2_, as previously described [35]. PCLSs were cultured up to 48 h and culture medium was refreshed every 24 h.

### 2.3. Culture Media

Williams medium E with Glutamax at pH 7.4 (Invitrogen, Bleiswijk, The Netherlands), supplemented with gentamycin (50mg/mL; Invitrogen) and L-carnitine (1 mM, Sigma-Aldrich, St. Louis, MO, USA), was used as control medium. To mimic metabolic syndrome, a culture medium with additional supraphysiological concentrations (Table 1, unless indicated otherwise) of glucose (Merck, Darmstadt, Germany), fructose (Merck), human insulin (Sigma-Aldrich), palmitic acid and oleic acid (Sigma-Aldrich) was used. Concentrations were based on human serum concentrations [36,37] and in vivo rodent portal vein concentrations [34,38] and optimised for this study.

Palmitic acid and oleic acid were dissolved in 0.1 M sodium hydroxide (Merck, Darmstadt, Germany) at 70 °C, and then mixed with preheated 0.04% BSA solution (Sigma-Aldrich, St. Louis, MO, USA) at 55 °C. The same concentration of BSA and sodium hydroxide was added to control media. Addition of this amount of BSA and sodium hydroxide did not affect medium pH and PCLS viability.

Sodium Butyrate (NaB; Sigma-Aldrich) and Sodium chloride (NaCl; Sigma-Aldrich) were dissolved in PBS (Gibco, ThermoFisher Scientific, Waltham, MA, USA). NaCl was used as a control for the NaB addition and is presented as NaCl throughout the paper to avoid confusion with the control medium CTR (Table 1). The concentration of NaB or NaCl in medium was 1mM, unless specified otherwise.

### 2.4. Triglyceride Quantitation

For each condition, three precision-cut liver slices were pooled, snap-frozen and stored at −80 °C. Subsequently, PCLSs were homogenized in 600 µL Tris-buffered saline (~25mg/mL) by bead beating for 30s at 5000 rpm with 4 to 5 2.3 mm zirconium beads (Precellys 24, Bertin Technologies, Montigny Le Bretonneux, France) at 4 °C. Fat was extracted using the Bligh & Dyer method [39]. In short, samples were mixed in a glass tube with 1500 µL of chloroform: methanol (2:1). Tubes were vortexed for 30 min at 4 °C. Subsequently, 600 µL of milliQ water and 500 µL of chloroform were added, samples were vortexed for 5 min and centrifuged at 1500× *g* for 10 min. Of the chloroform layer, 900 µL were moved into a new glass tube and dried under N_2_ stream. Lipids were dissolved in 150 µL of 2% triton-X in chloroform, dried under N_2_ and dissolved in 150 milliQ µL water while shaking at 37 °C in a water bath for 30 min. Triglyceride content was determined using a Trig/GB kit (Roche Molecular Biochemicals, 11877771, Almere, The Netherlands) according to the protocol provided by the manufacturer. Values were calculated using a glycerol standard curve and normalized for protein content using the Pierce BCA Protein Assay Kit (ThermoFisher Scientific). Data are presented as percentage of the control (CTR) ± SEM.

### 2.5. Acylcarnitine Measurement

Acylcarnitines are measured as a proxy for β-oxidation. For each condition, three precision-cut liver slices were pooled, snap-frozen and stored at −80 °C. Subsequently, PCLSs were homogenized in 600 µL tris-buffered saline (~25 mg/mL) by bead beating for 30 s at 5000 rpm with 4 to 5 2.3 mm zirconium beads at 4 °C. In brief, 250 µL of cold acetonitrile was added to 25 µL of sample, shortly vortexed and then 250 µL of internal standard (L-carnitine-[D_3_], acetyl-L-carnitine-[D_3_], propionyl-L-carnitine-[D_3_], octanoyl-L-carnitine-[D_3_], and palmitoyl-L-carnitine-[D_3_]) was added to the mix. Subsequently, samples were vortexed again and centrifuged (RT, max speed 20,000× *g*) for 10 min. Finally, 150 µL from each sample were transferred to an injection vial with insert for LC-MS/MS analysis. Acylcarnitine quantification was normalized for protein content using the Pierce BCA Protein Assay Kit.

### 2.6. ATP Determination

Intracellular ATP content was measured to indicate viability. For each condition, individual precision-cut liver slices were collected in 1 mL of SONOP buffer (2 mM EDTA in 70% ethanol at pH 10.9), snap-frozen and stored at −80 °C. ATP content was measured using a bioluminescence kit (Roche Diagnostics, Mannheim, Germany) as previously described [35]. Measured ATP was corrected for total protein content using a Lowry assay (BioRad DC Protein Assay, Hercules, CA, USA). Values for each group were expressed as a (Δ) percentage of the 48 h control (CTR or NaCl) ± SEM.

### 2.7. Release of Pro-Collagen Iα1

As a measure of fibrosis, release of pro-collagen Iα1 was measured. Pooled culture medium from three PCLSs per condition per mouse was frozen at −20 °C and diluted tenfold. Levels of excreted protein were measured using the mouse Pro-Collagen I alpha 1 kit from (ab210579; Abcam, Cambridge, UK) according to instructions from the manufacturer. Values were normalized for total protein content using the Pierce BCA Protein Assay Kit.

### 2.8. Quantitative Real-Time PCR

To measure mRNA levels, three PCLSs were pooled per condition, snap-frozen in liquid nitrogen, and stored at −80 °C. RNA was isolated using the RNeasy Lipid Tissue Mini Kit (Qiagen, Venlo, The Netherlands). The Reverse Transcription System (Promega, Leiden, The Netherlands) was used to reverse transcribe RNA. cDNA was amplified using SYBR Green Mixture (Bio-rad Laboratories, Veenendaal, The Netherlands). Primer sequences are displayed in Supplementary Table S1 (ThermoFisher Scientific). Gene symbols are presented in line with MGI guidelines.

Quantitative real-time PCR was performed using a QuantStudio™ 7 Flex System (ThermoFisher Scientific). Thermal cycling started with a 10 min hold at 95 °C, followed by 40 cycles that consisted of 15 s at 95 °C, 30 s at 60 °C, and 30 s at 72 °C. Ct values were corrected for the Ct values of *Hmbs*, which has previously been identified as a stable marker in the murine liver [40]. This yielded ΔCt values that were used to perform statistics and calculate −ΔΔCt values. Z-scores are used to present differential expression in heat maps (*n* = 5).

### 2.9. Statistics

Every experiment was performed at least as a biological triplicate, i.e., with PCLSs obtained from three different mice. Results are expressed as means ± standard error of the mean (SEM) and compared to the control group. Individual values are shown as circles. Comparisons were made using a paired one-way ANOVA with Dunnett’s post hoc analysis when GFI or GFIPO were compared to CTR medium, and a paired two-way ANOVA with Šídák’s multiple comparison analysis when NaB was compared to NaCl (CTR, GFI or GFIPO). For the heat maps with only two groups a paired *t*-test was performed. Results were considered statistically significant when the calculated *p*-value was smaller than 0.05.

## 3. Results

### 3.1. Induction and Characterisation of Steatosis in PCLSs

To study the direct effect of butyrate on early-stage MAFLD, this phenotype was first induced in healthy murine precision-cut liver slices. Culture of PCLSs with concentrations of 36 mM glucose, 5 mM fructose, 240 µM palmitate, 480 µM oleate and 100 nM insulin (High-GFIPO; as previously used in rat PCLSs [34]), resulted in a 2-fold increase in triglyceride content after 48 h compared to the control medium (Supplementary Figure S1A). Besides the induction of steatosis, this condition substantially reduced intracellular ATP content, a measure of viability (Supplementary Figure S1B), possibly indicating that the quality of PCLSs deteriorated.

To induce steatosis and preserve viability with the minimum amount of energy substrates added, the concentrations of sugars and fatty acids were varied. There was no significant difference in triglyceride accumulation when the glucose concentration was decreased from the previously used 36 mM to 11 mM (Supplementary Figure S1A). After fructose titration, 1 mM fructose was sufficient to achieve the maximum TG accumulation after 24 and 48 h (Supplementary Figure S2A,B). While the significance of TG accumulation did not depend on the concentration of palmitate and oleate, the accumulation was most reproducible when using 120 µM and 240 µM, respectively (Supplementary Figure S2A,B). Moreover, fat accumulation was more reproducible after 48 h especially with 1mM fructose, thus PCLSs were cultured for 48 h in subsequent experiments.

**Figure 1:**
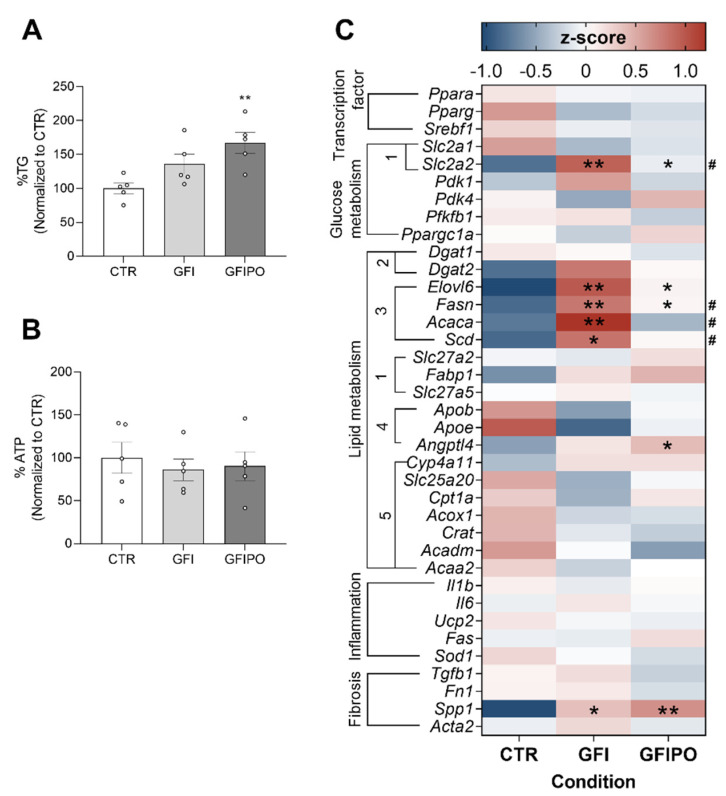
Induction and characterisation of steatosis in PCLS. (**A**) TG levels and (**B**) ATP concentration after 48 h incubation with GFI or GFIPO medium, compared to CTR. Data are presented as mean percentage ± SEM. (**C**) Heat map of mRNA levels of genes encoding transcription factors, transporters (1) and enzymes (2: triglyceride assembly, 3: lipid synthesis, 4: FA efflux, 5: FA oxidation) involved in glucose and fatty acid metabolism, inflammation and fibrosis after 48 h incubation in CTR, GFI or GFIPO medium. Medium compositions were abbreviated according to Table 1 (CTR = control; GFI = glucose, fructose, and insulin; and GFIPO = GFI plus palmitic acid and oleic acid). Differential expression is presented using Z-scores (red: high Z-score, upregulated; blue: low Z-score, downregulated). * = *p* < 0.05, ** = *p* < 0.01 compared to CTR, # = *p* < 0.05 compared between GFI and GFIPO. Individual values are shown as circles.

After 48 h of incubation in the presence of glucose, fructose, insulin, palmitate and oleate (GFIPO, concentrations in Table 1), triglyceride levels were increased by 67% as compared to the control (Figure 1A). The significant increase in C14-C18 acylcarnitines confirmed that the supplied palmitate and oleate were taken up by PCLSs (Supplementary Table S2). To be able to differentiate between the effect of fatty acid influx and the effect of de novo lipogenesis, a condition with additional fructose (5 mM) and no fatty acids (GFI, concentrations in Table 1) was added. This medium caused a smaller accumulation of triglycerides as compared to GFIPO medium (36%, Figure 1A), suggesting that the fatty acid uptake makes a large contribution to the TG accumulation. Neither culture medium altered ATP significantly, indicating that these optimized steatosis-inducing media did not affect PCLSs viability (Figure 1B).

Compared to the condition with only glucose as an energy substrate (CTR), both the GFI and the GFIPO medium upregulated mRNA levels of genes encoding proteins involved in fatty acid synthesis and hexose uptake, namely elongation of very long chain fatty acids protein 6 (*Elovl6*), fatty acid synthase (*Fasn*), and glucose transporter 2 (*Slc2a2*) (Figure 1C, clusters 1 and 3). The condition with a higher fructose concentration, but no fatty acids (GFI) had a bigger impact on these genes, and additionally upregulated expression of acetyl-CoA carboxylase (*Acaca*) and Acyl-CoA desaturase (*Scd*) (Figure 1C, cluster 3). Besides, there was a marked decrease of genes encoding enzymes involved in oxidation of fatty acids, when they were analysed as a panel. The reduction of the cluster as a whole was significant with a *p*-value of <0.05 for GFIPO (Figure 1C, cluster 5). These data suggest that induction of steatosis in PCLSs relies also on de novo synthesis of lipids, more so in GFI than in GFIPO medium, together with reduced fatty acid catabolism.

Inflammation and fibrosis play an important role in the progression of MAFLD. Therefore, gene expression of markers of an inflammatory response and tissue remodelling were measured. Interestingly, neither medium affected inflammation at the mRNA level. Media did cause an increased gene expression of osteopontin (*Spp1*), a marker of fibrosis [41], indicating that there might be tissue remodelling and fibrosis. In conclusion, both GFI and GFIPO media induce steatosis but do not instigate an inflammatory response at the mRNA level.

### 3.2. Effects of Butyrate Treatment on Fatty Acid and Triglyceride Synthesis

**Figure 2:**
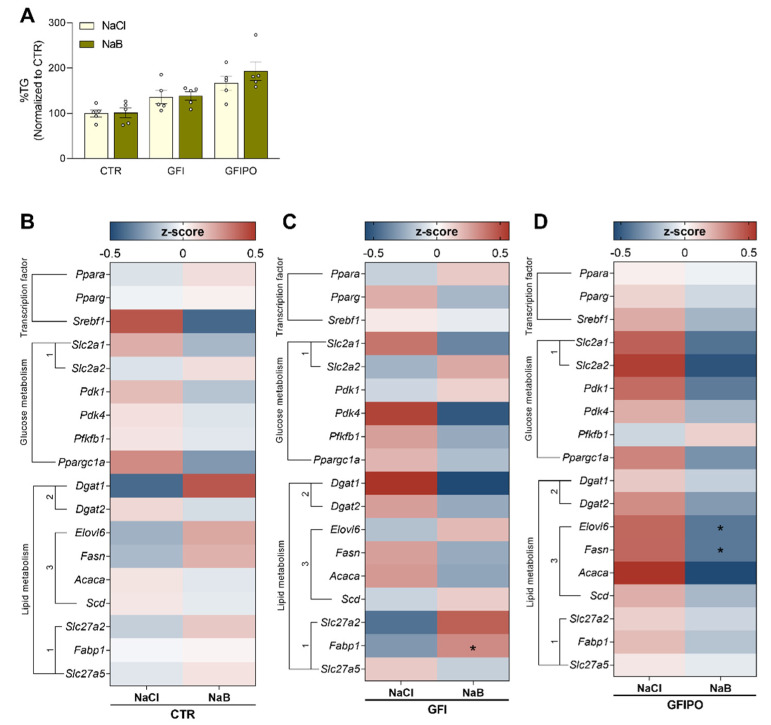
Effects of butyrate treatment on triglyceride accumulation and metabolism. (**A**) Relative TG levels in the three different media with 1 mM NaCl (control) or 1mM NaB (sodium butyrate). Data are presented as mean percentage of control ± SEM. (**B**–**D**) Heat maps of measured mRNA expression levels of genes related to lipid and glucose metabolism after culture in (**B**) CTR medium, (**C**) GFI medium, (**D**) GFIPO medium with 1 mM NaCl or 1mM NaB. Medium compositions were abbreviated according to Table 1 (CTR = control; GFI = glucose, fructose, and insulin; and GFIPO = GFI plus palmitic acid and oleic acid). Differential expression is presented using Z-scores (red: high Z-score, upregulated; blue: low Z-score, downregulated). * = *p* < 0.05 compared to NaCl. Individual values are shown as circles.

Butyrate has been found to decrease hepatic fat accumulation in vivo [10]. To test whether this could be a direct effect of butyrate, it was assessed whether butyrate prevented triglyceride accumulation when PCLSs were incubated with sugars and lipids. Triglyceride content was not affected by 1 mM butyrate in either GFI or GFIPO medium (Figure 2A). A higher concentration of butyrate (3 mM) rather tended to increase TG levels (Supplementary Figure S3A). Transcript levels of genes involved in glucose metabolism and lipid synthesis were not regulated by butyrate in the control medium (Figure 2B). In GFI medium fatty acid binding protein 1 (*Fabp1*), involved in hepatic fatty acid transport and metabolism [42], was increased by butyrate (Figure 2C). In GFIPO medium, mRNAs encoding proteins in fatty acid synthesis, *Elovl6* and *Fasn*, were significantly reduced by butyrate (Figure 2D). These were among the genes that were upregulated by GFIPO medium originally.

This suggests that in GFIPO butyrate tended to downregulate fatty acid synthesis, but the effect may have been overruled by the overload of substrates, including the butyrate itself. This could explain why TG levels were not affected by butyrate, while fatty acid synthesis genes were downregulated.

### 3.3. Effects of Butyrate Treatment on Clearance and Oxidation of Fatty Acids

**Figure 3:**
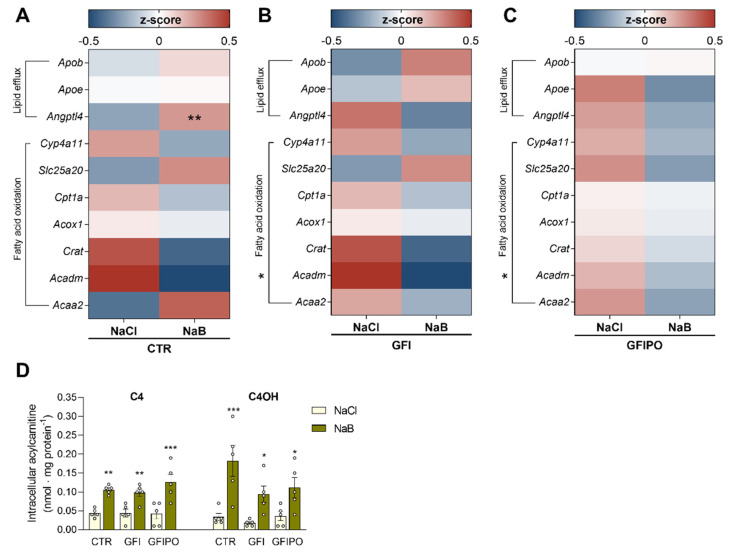
Effects of butyrate treatment on clearance and oxidation of fatty acids. Heat map of measured expression levels of genes related to lipid efflux (*Apob*, *Apoe and Angptl4*) and genes related to fatty acid oxidation (*Slc25a20, Pdk4, Cyp4a11, Crat, Acox1, Acadm* and *Cpt1*) after culture in (**A**) CTR medium, (**B**) GFI medium, (**C**) GFIPO medium with 1 mM NaCl (control) or 1 mM NaB (butyrate). Medium compositions were abbreviated according to Table 1 (CTR = control; GFI = glucose, fructose, and insulin; and GFIPO = GFI plus palmitic acid and oleic acid). Differential expression is presented using Z-scores (red: high Z-score, upregulated; blue: low Z-score, downregulated). (**D**) Intracellular butyrylcarnitine (C4) and hydroxybutyrylcarnitine (C4OH) quantification in CTR, GFI and GFIPO with 1 mM NaCl or 1 mM NaB. Data are presented as mean ± SEM. * = *p* < 0.05, ** = *p* < 0.01, *** = *p* < 0.001 compared to NaCl. Individual values are shown as circles.

To investigate the effects of butyrate on lipid clearance and oxidation, mRNA levels of genes involved in these processes were measured (Figure 3). Minor differences were seen for genes involved in lipid efflux. While apolipoprotein expression remained unaffected by butyrate in all conditions, mRNA levels of the lipoprotein lipase activity mediator *Angptl4* were significantly increased in CTR medium (Figure 3A), and slightly reduced in GFI and GFIPO medium (Figure 3B,C).

Butyrate did not exert significant effects on any of the individual genes coding for enzymes involved in fatty acid oxidation, although a trend of downregulation was visible at the level of the entire cluster. When these genes were assessed as a panel, treatment with butyrate resulted in a significant reduction in GFI and GFIPO medium (*p* < 0.05, Figure 3B,C).

Since fatty acids are esterified to carnitine before they are oxidized, acylcarnitine concentrations may be considered as a proxy for ongoing β-oxidation. To functionally assess β-oxidation, the profile of acylcarnitines was measured in the PCLSs. Long-chain fatty acids such as palmitate (C16:0) and oleate (C18:1) are activated and converted to acylcarnitines by carnitine palmitoyltransferase 1 (CPT1) to be able to enter the mitochondrial β-oxidation pathway [43]. Palmitate and oleate addition in the GFIPO condition increased the overall carnitine profile (Supplementary Figure S3B and Supplementary Table S2). Butyrate specifically increased butyrylcarnitine (C4) and hydroxybutyrylcarnitine (C4OH) in all conditions, but did not alter the long-chain acylcarnitines. This demonstrates that butyrate is metabolized in the PCLSs, but does not affect the oxidation of long-chain fatty acids (Figure 3D, Supplementary Figure S3B and Supplementary Table S2). Acetylcarnitine (C2) levels were not significantly changed by either medium and only had a trend to increase with butyrate (Supplementary Figure S3C). Together the gene-expression and acylcarnitine levels show that butyrate is metabolised by the PCLSs, but does not affect long-chain fatty acid oxidation nor lipid efflux.

### 3.4. The Effect of Butyrate on Inflammation and Tissue Remodelling

**Figure 4:**
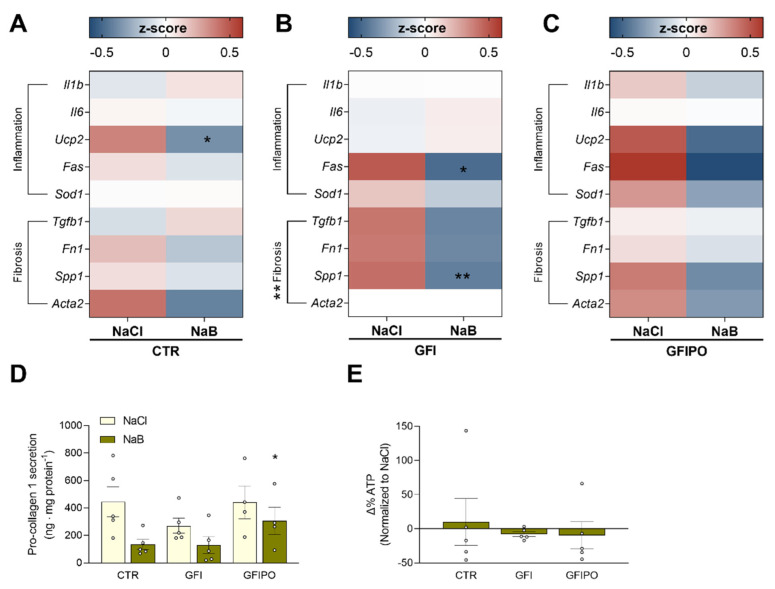
Effects of butyrate on inflammation and tissue remodelling. (**A**–**C**) Heat maps displaying mRNA expression of genes indicative of inflammation (*Il1b*, *Il6*, *Ucp2*, *Fas*, *Sod1*) and genes related to tissue remodelling (*Tgfb1*, *Fn1*, *Spp1*, *Acta2*) after culture in (**A**) CTR medium, (**B**) GFI medium, (**C**) GFIPO medium with 1 mM NaCl (control) or 1 mM NaB (butyrate). Differential expression is presented using Z-scores (red: high Z-score, upregulated; blue: low Z-score, downregulated). (**D**) Excreted pro-collagen Iα1 after culture in CTR, GFI and GFIPO medium with 1mM NaCl or 1mM NaB. (**E**) Difference in intracellular ATP content after culture with NaB as compared to NaCl. Medium compositions were abbreviated according to Table 1 (CTR = control; GFI = glucose, fructose, and insulin; and GFIPO = GFI plus palmitic acid and oleic acid). Data are presented as mean ± SEM.* = *p* < 0.05, ** = *p* < 0.01 compared to NaCl. Individual values are shown as circles.

Since inflammation and fibrosis play a crucial role in the progression of MAFLD, the effects of butyrate on these processes were examined (Figure 4A–C). Both UCP2 and SOD1 are known to play a role in lipid metabolism as well as oxidative stress, and are linked to the development of NASH [44,45]. Butyrate treatment resulted in a trend of downregulation of *Ucp2*, *Sod1*, and death receptor *Fas* (not to be confused with fatty-acid synthase *Fasn*), but not of interleukins 1β and 6 which are more specific for inflammation. Differences in the regulation of fibrosis genes were more pronounced. Butyrate reduced the expression of fibrosis-related mRNAs in all media (with *p*-values of <0.18, 0.01 and 0.07 for CTR, GFI and GFIPO, respectively) (Figure 4A–C). These changes in fibrotic markers were confirmed by pro-collagen Iα1 secretion, a functional measure of fibrosis (Figure 4D). This secretion was not significantly altered by the GFI or GFIPO media. Butyrate, however, lowered the levels of pro-collagen Iα1 in all conditions, up to an average reduction of 67 ± 14% in CTR culture medium. Viability, assessed as ATP content, was not affected by butyrate (Figure 4E). Together, these data suggest that fibrosis in PCLCs is reduced by butyrate supplementation.

## 4. Discussion

To study MAFLD, the PCLS model is a promising tool that recapitulates the multicellular architecture of the liver. This technique supports the 3Rs of Replacement, Reduction and Refinement of animal use [46,47], since multiple conditions can be tested in the same liver. In this paper, steatosis was first induced in healthy murine liver slices. The minimum concentrations of glucose, fructose, insulin and fatty acids that resulted in robust induction of steatosis without loss of viability were identified. Steatosis could be attributed to a combination of *de novo* lipogenesis, fatty acid uptake and reduced fatty acid oxidation. Butyrate did not prevent steatosis, but reduced fibrosis, an important characteristic of MAFLD progression.

Overconsumption of sugars and fat contributes to hepatic steatosis [8,48,49]. Fructose is particularly lipogenic, since in contrast to glucose, it is metabolized by the liver in an unregulated fashion [50,51]. The products pyruvate and acetyl-CoA are used as substrates for *de novo* lipogenesis, which is increased in MAFLD patients [8,52]. In addition, dietary long-chain fatty acids, such as palmitate and oleate, can be elongated and stored in the liver as triglycerides [53,54]. In this study, steatosis was induced by supplementation of 11 mM glucose, 1 mM fructose, 100 nM insulin and a mixture of 0.36 mM palmitate and oleate (GFIPO) and by the omission of fatty acids while increasing to 5 mM fructose (GFI). In comparison, fructose plasma concentrations depend strongly on diet but can reach well over 5 mM (humans) and 1 mM (rodents) [38,55], and the free fatty acid concentration up to 1 mM [56,57,58]. These may be even higher in the portal vein, as indicated by previous measurements in rodents [38,59]. Since fructose is a substrate for de novo lipogenesis [8], it is not surprising that the GFI medium elicited the highest upregulation of lipogenic gene expression. Progression of human MAFLD is characterized by inflammation and fibrosis [3,60]. Fibrosis could be attributed to palmitate-related lipotoxicity [61] or fructose-related ATP depletion [62]. No inflammatory response was measured in PCLSs, but this could possibly be instigated by addition an inflammatory stimulus like lipopolysaccharide, which is elevated in MAFLD patients [63,64]. The fact that the PCLSs showed a fibrotic response, but no inflammatory response, suggests that they mimic early-stage MAFLD.

In vivo supplementation of butyrate or butyrate-producing bacteria alleviate features of metabolic syndrome, including hepatic steatosis and fibrosis [11,12,13,14,15,16]. Butyrate can act as a regulator, inducing fatty acid oxidation and reducing lipogenesis [11,14], but it is also a precursor for *de novo* lipogenesis as shown by the administration of labelled butyrate [26]. The use of PCLSs allowed to investigate the direct effect on liver tissue, as opposed to the indirect effects via cross-talk with other organs. The concentrations used in this study were in the high physiological range (1 mM), to avoid depletion due to metabolism. In comparison, plasma butyrate concentrations are typically in the micromolar range [20,65,66]. However, plasma butyrate reached transient mM concentrations in mice that were fed with butyrate [67] and again, portal vein concentrations could be higher, but measurements are scarce. In agreement with in vivo studies [26,68], we found that butyrate served as a metabolic substrate, inferred here by the elevated C4 and C4OH carnitine levels in the butyrate groups. This could lead to an increase in acetyl-CoA, which may explain why butyrate did not decrease, but rather increased TG accumulation in the PCLSs. Moreover, in contrast to in vivo studies [11] and HepG2 cells [24], butyrate reduced mRNA levels of proteins involved in fatty acid oxidation in PCLSs. The mRNA levels of lipogenesis genes *Elovl6* and *Fasn* were decreased by butyrate only in the GFIPO and not in the GFI condition. The counteracting effects of butyrate as a regulator and as a substrate for lipogenesis is of high interest when developing butyrate as an intervention for metabolic disease. The fact that butyrate elicits a decrease in steatosis in vivo [11], but not in PCLSs, suggests that the regulatory role of butyrate can be a consequence of inter-organ crosstalk. Reported effects of butyrate include decreased lipolysis in the adipose tissue which prevents the excess of lipid supply to other organs [10,69] and increased GLP-1 and PYY secretion from the gut, which regulates food intake and satiety [10,17].

In mice with advanced MAFLD, butyrate reduced the mRNA levels of inflammatory genes *Ilb* and *Il6* [15,16,70]. That this was not recapitulated in PCLSs, may be attributed to the fact that inflammation was anyway low in this in vitro model. The difference between in vivo results and PCLSs may be due to the absence of circulating immune cells, such as monocytes and infiltrating macrophages, that present G-protein coupled receptors that can bind butyrate [70,71]. Therefore, the in vivo effects might have derived from organ interplay, and not from direct liver effects. In contrast, the PCLSs exhibited signs of fibrosis, a typical feature of MAFLD [3]. These were alleviated by butyrate. The key players in liver fibrosis are hepatic stellate cells (HSCs) [72], which produce α-smooth muscle actin (αSMA; *Acta2*) and other extracellular matrix components. Limited studies confirm that butyrate can attenuate rat HSC activation, thereby lowering the expression of collagens and αSMA [73]. This effect is seen in vivo and in fresh primary HSCs, but understandably not in HepG2 cells as they cannot produce collagen [14,74]. The presence of HSCs in PCLSs [75] may explain the anti-fibrotic effects of butyrate in this study.

## 5. Conclusions

In conclusion, murine PCLSs treated with insulin, carbohydrates and fatty acids recapitulate key features of early-stage MAFLD, making them a promising ex vivo model for the disease. The intact tissue structure of the precision-cut liver slices makes them particularly a good model to study mechanisms underlying steatosis, fibrosis and potential treatments. The translational value of this model would be even greater when human liver tissue is used. In the present ex vivo model butyrate did not reduce steatosis, suggesting that butyrate was mostly used as a substrate for lipogenesis under the investigated culture conditions. These contradictory effects of butyrate will be of relevance for clinical studies in which liver concentrations of butyrate are increased.

## Figures and Tables

**Table 1:** Composition of culture media.

Medium	Additives	Final Concentration in Medium				
		Glucose	Fructose	Insulin	Palmitic Acid	Oleic Acid
CTR	Glucose	11 mM				
GFI	Glucose, fructose, and insulin	11 mM	5 mM	1 nM		
GFIPO	Glucose, fructose, insulin, palmitic acid and oleic acid	11 mM	1 mM	1 nM	120 μM	240 μM

CTR = control; GFI = glucose, fructose and insulin; GFIPO = glucose, fructose, insulin, palmitic acid and oleic acid.

## Data Availability

Not applicable.

## Supplementary Materials

The following are available online at https://www.mdpi.com/article/10.3390/nu13124203/s1, Figure S1: Investigation of substrate additives in PCLSs, Figure S2: The effect of fructose and fatty acid supplementation on triglyceride accumulation, Figure S3: The effect of butyrate on triglyceride accumulation and acylcarnitine profile, Table S1: List of oligonucleotide primer pairs used in qPCR analysis, Table S2: Acylcarnitine quantification.
