# Supplementary material for: The Effects of Butyrate on Induced Metabolic-Associated Fatty Liver Disease in Precision-Cut Liver Slices
Source: Nutrients. 2021 Nov 24;13(12):4203. doi: 10.3390/nu13124203 (PMC8703944; doi:10.3390/nu13124203)
Supplement: Supplementary file 1 [file nutrients-13-04203-s001.zip › nutrients-1440380-supplementary.pdf]

Supplementary figures

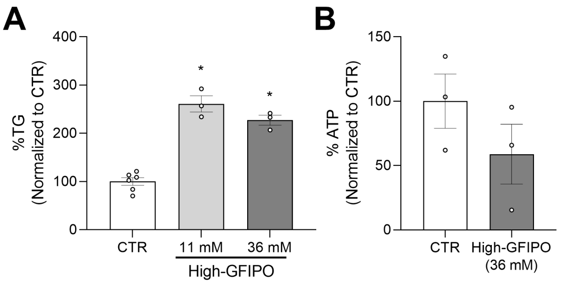

Supplementary Figure S1. Investigation of substrate additives in PCLS. (A-B) Incubation of PCLS in presence of 240 µM palmitate, 480 µM oleate, 5 mM fructose, 1 nM insulin (High-GFIPO) with 11 mM and 36 mM glucose and CTR medium (CTR medium as described in Table 1). (A) Relative TG levels after incubation in High-GFIPO with 11 or 36mM glucose and (B) intracellular ATP content with High-GFIPO with 36 mM glucose. All data are presented as mean percentage ± SEM. * = p < 0.05 compared to the relative control condition.

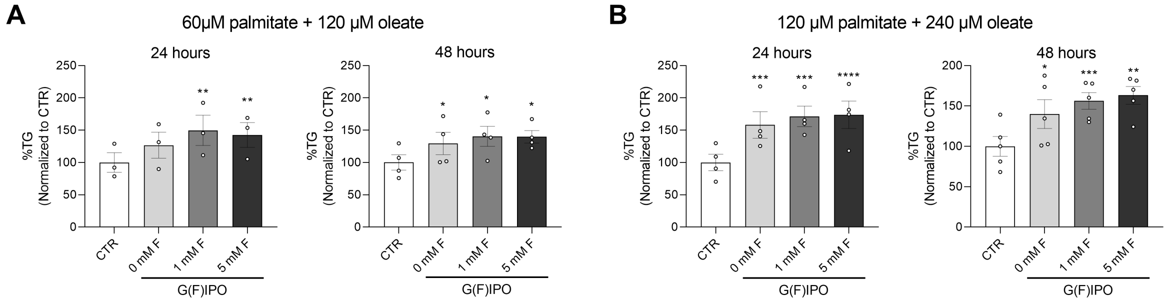

Supplementary Figure S2. The effect of fructose and fatty acid supplementation on triglyceride accumulation. PCLS were incubated for 24 and 48 hours with 11 mM glucose, 1 nM insulin and varying levels of fructose, palmitate and oleate or CTR medium (CTR medium as described in Table 1). (A) Relative TG levels after incubation of PCLS in presence of 60 µM palmitate, 120 µM oleate, and 1-5 mM fructose. (B) Relative TG levels after incubation of PCLS in presence of 120 µM palmitate, 240 µM oleate, and 1-5 mM fructose. All data are presented as mean ± SEM. * = p < 0.05, ** = p < 0.01, *** = p < 0.001, **** = p < 0.0001 compared to CTR.

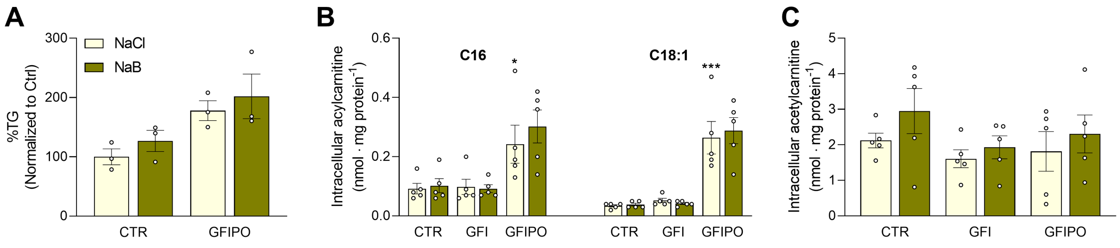

Supplementary Figure S3. The effect of butyrate on triglyceride accumulation and acylcarnitine profile. (A) Relative TG levels after 48 hours incubation with 3mM NaCl (control) or 3mM NaB (butyrate) in CTR or GFIPO medium. (B) Intracellular palmitoylcarnitine (C16), oleoylcarnitine (C18:1) and (C) acetylcarnitine (C2) after incubation in CTR, GFI and GFIPO with 1 mM NaCl or 1 mM NaB. All media compositions according to Table 1. All data are presented as mean ± SEM. * = p < 0.05, *** = p < 0.001 compared to relative control condition.

Supplementary tables

Supplementary Table S1. List of oligonucleotide primer pairs used in qPCR analysis.

Gene	Encodes for	Primer sequence Forward (5′-3′)	Reverse (5′-3′)
Acaa2	ACAA2	CTGCTACGAGGTGTGTTCATC	AGCTCTGCATGACATTGCCC
Acaca	ACC1	GCGTCGGGTAGATCCAGTT	CTCAGTGGGGCTTAGCTCTG
Acadm	MCAD	GCTAGTGGAGCACCAAGGAG	CCAGGCTGCTCTCTGGTAAC
Acadm	MCAD	AGGGTTTAGTTTTGAGTTGACGG	CCCCGCTTTTGTCATATTCCG
Acox1	ACOX	ATGCCTTTGTTGTCCCTATC	CCATCTTCAGGTAGCCATTATC
Acta2	ASMA	ACTACTGCCGAGCGTGAGAT	CCAATGAAAGATGGCTGGAA
Angptl4	ANGPTL4	GGGACCTTAACTGTGCCAAG	GAATGGCTACAGGTACCAAACC
Apob	APOB	AAGCACCTCCGAAAGTACGTG	CTCCAGCTCTACCTTACAGTTGA
Apoe	APOE	CTGACAGGATGCCTAGCCG	CGCAGGTAATCCCAGAAGC
Cpt1	CPT1A	CTCCGCCTGAGCCATGAAG	CACCAGTGATGATGCCATTCT
Crat	CRAT	GCTGCCAGAACCGTGGTAAA	CCTTGAGGTAATAGTCCAGGGA
Cyp4a11	CYP4A11	GCTAGCTCCTTGGATTGGGTA	AGGGTTTCAGAATGTCATAGTGG
Dgat1	DGAT1	TCCGTCCAGGGTGGTAGTG	TGAACAAAGAATCTTGCAGACGA
Dgat2	DGAT2	GCGCTACTTCCGAGACTACTT	GGGCCTTATGCCAGGAAACT
Elovl6	ELOVL6	GAAAAGCAGTTCAACGAGAACG	AGATGCCGACCACCAAAGATA
Fabp1	FABP1	ATGAACTTCTCCGGCAAGTACC	CTGACACCCCCTTGATGTCC
Fasn	FASN	CTGCGGAAACTTCAGGAAATG	GGTTCGGAATGCTATCCAGG
Fas	FAS	TATCAAGGAGGCCCATTTTGC	TGTTTCCACTTCTAAACCATGCT
Fn1	FN1	CGGAGAGAGTGCCCCTACTA	CGATATTGGTGAATCGCAGA
Hmbs	HMBS	ATGAGGGTGATTCGAGTGGG	TTGTCTCCCGTGGTGGACATA
Il1b	IL1B	GCCAAGACAGGTCGCTCAGGG	CCCCCACACGTTGACAGCTAGG
Il6	IL6	TGATGCTGGTGACAACCACGGC	TAAGCCTCCGACTTGTGAAGTGGTA
Mttp	MTTP	CTCTTGGCAGTGCTTTTTCTCT	GAGCTTGTATAGCCGCTCATT
Pdk1	PDK1	GGACTTCGGGTCAGTGAATGC	TCCTGAGAAGATTGTCGGGGA
Pdk4	PDK4	TTGACTCCCACATTGGTTGA	TCATGTGCACAAACTCAGAGC
Pfkfb1	PFKFB1	ATGAGCTGCCCTATCTCAAGT	GTCCCGGTGTGTGTTCACAG
Ppara	PPARA	AGAGCCCCATCTGTCCTCTC	ACTGGTAGTCTGCAAAACCAAA
Pparg	PPARG	TCGCTGATGCACTGCCTATG	GAGAGGTCCACAGAGCTGATT
Ppargc1a	PGC1α	GACATAGAGTGTGCTGCTCTG	ATTGGTCGCTACACCACTTC
Scd	ACOD1	TTCTTGCGATACACTCTGGTGC	CGGGATTGAATGTTCTTGTCGT
Slc25a20	CACT	GACGAGCCGAAACCCATCAG	AGTCGGACCTTGACCGTGT
Slc27a2	FATP2	TCCTCCAAGATGTGCGGTACT	TAGGTGAGCGTCTCGTCTCG

Slc27A5	FATP5	CTACGCTGGCTGCATATAGATG	CCACAAAGGTCTCTGGAGGAT
Slc2a1	GLUT1	TTCTCTGTCGGCCTCTTTGT	GAGAAGCCCATAAGCACAGC
Slc2a2	GLUT2	TCAGAAGACAAGATCACCGGA	GCTGGTGTGACTGTAAGTGGG
Sod1	SOD1	AACCAGTTGTGTTGTCAGGAC	CCACCATGTTTCTTAGAGTGAGG
Spp1	OPN	AGCAAGAAACTCTTCCAAGCAA	GTGAGATTCGTCAGATTCATCCG
Srebf1	SRBP1	TGACCCGGCTATTCCGTGA	CTGGGCTGAGCAATACAGTTC
Tgfb1	TGF-β1	CCGAATGTCTGACGTATTGAAGA	GCGGACTACTATGCTAAAGAGG
Ucp2	UCP2	ATGGTTGGTTTCAAGGCCACA	CGGTATCCAGAGGGAAAGTGAT

Supplementary Table S2. Acylcarnitine quantification.

	CTR				GFI				GFIPO			
	NaCl		NaB		NaCl		NaB		NaCl		NaB	
Acylcarnitine	38.89	± 2.56	30.20	± 1.83	30.59	± 2.78	29.24	± 1.28	33.39	± 4.05	34.59	± 2.84
C0	36.13	± 2.47	$26.50	± 1.27	28.41	± 2.55	26.66	± 1.12	30.47	± 3.47	30.84	± 2.22
C2	2.12	± 0.21	2.95	± 0.64	1.61	± 0.25	1.93	± 0.32	1.81	± 0.56	2.31	± 0.53
C3	0.08	± 0.01	$0.03	± 0.01	0.05	± 0.01	0.04	± 0.01	0.06	± 0.02	0.04	± 0.01
C4	0.04	± 0.01	$$0.11	± 0.01	0.04	± 0.01	$$0.10	± 0.01	0.04	± 0.01	$$$0.12	± 0.022
C5	0.03	± 0.00	$0.01	± 0.00	0.02	± 0.00	0.01	± 0.00	0.02	± 0.01	0.01	± 0.00
C6	0.00	± 0.00	0.00	± 0.00	0.00	± 0.00	0.00	± 0.00	0.01	± 0.00	0.01	± 0.00
C8	nd		nd		nd		nd		nd		nd	
C10	nd		nd		nd		nd		nd		nd	
C12:1	nd		nd		nd		nd		nd		nd	
C12	nd		nd		nd		nd		0.00	± 0.00	0.00	± 0.00
C14:1	nd		nd		nd		nd		0.00	± 0.00	$$0.01	± 0.00
C14	0.02	± 0.00	0.02	± 0.00	0.02	± 0.00	0.02	± 0.00	**0.06	± 0.01	0.07	± 0.01
C16:1	0.00	± 0.00	0.01	± 0.00	0.01	± 0.00	0.01	± 0.00	**0.04	± 0.01	0.04	± 0.01
C16	0.09	± 0.02	0.10	± 0.02	0.10	± 0.03	0.09	± 0.01	*0.24	± 0.06	0.30	± 0.06
C18:2	0.01	± 0.00	0.01	± 0.00	0.01	± 0.00	0.01	± 0.00	**0.03	± 0.01	0.03	± 0.01
C18:1	0.04	± 0.00	0.04	± 0.00	0.05	± 0.01	0.04	± 0.00	***0.27	± 0.05	0.29	± 0.04
C18	0.07	± 0.01	0.08	± 0.02	0.09	± 0.02	0.08	± 0.01	0.12	± 0.02	0.15	± 0.02
C4OH/C3DC	0.03	± 0.01	$$$0.18	± 0.04	0.02	± 0.00	$0.09	± 0.02	0.03	± 0.01	$0.11	± 0.03
C5OH/C4DC	nd		nd		nd		nd		0.00	± 0.00	nd	

*Values are presented as mean ± SEM in nmol/mg protein, nd = not detected. * = $p < 0.05$, ** = $p < 0.01$, *** = $p < 0.001$ comparing GFI (NaCl) and GFIPO (NaCl) to CTR (NaCl), and \$ = $p < 0.05$, \$\$ = $p < 0.01$, \$\$\$ = $p < 0.001$ comparing NaB to NaCl in the same medium.*
